# 

**Published:** 1889-12

**Authors:** 


CONTENTS

Vol. 36 No. 12

| | PAGE. |
|---|---|
| Looking Forward | 265 |
| Looking Forward More Truly | 270 |
| The Chapin Home | 271 |
| Fingers Before Forks | 272 |
| Proper Food for Consumptives | 273 |
| Marriage and Birth Statistics in England | 274 |
| Rudimentary Organs | 275 |
| Psychism | 276 |
| Christian Science | 277 |
| Paints and Dyes | 278 |
| The N. Y. Court of Appeals on Adulteration | 279 |
| Child Whipping | 280 |
| A Cold and its Treatment | 280 |
| Measles | 280 |
| Tokology | 281 |
| Public Hot Baths in Chin | 282 |
| Gymnasiums for Girls | 283 |
| Sunflowers and Malaria | 284 |
| Whither are we Drifting | 285 |
| A Drop of Water | 285 |
| Household | 286 |
| Miscellaneous | 287 |
| Literary | 288 |

INDEX TO ADVERTISEMENTS OF HALF A PAGE AND OVER.

| | PAGE |
|---|---|
| Consumers Supply Association | I |
| Celluloid | II |
| Hollow Suppositories | III |
| Christ Before Pilate | IV |
| Lactopeptine | V |
| E. C. Morris & Co | VI |
| Dennin's Certain Cure | VII |
| An Unprecedented Offer | VIII |
| The Herb Cure Co | X |
| Revised Premium List | XI |

